# Rural Identity and LGBT Public Opinion in the United States

**DOI:** 10.1093/poq/nfad045

**Published:** 2023-11-03

**Authors:** Jack Thompson

**Affiliations:** Postdoctoral Research Fellow, Department of Politics, University of Exeter, Exeter, UK

## Abstract

Opposition to LGBT rights remains a contemporary fixture within the United States in spite of increasingly liberalizing attitudes toward LGBT individuals. In this paper, I argue that a potentially overlooked factor driving this opposition is rural identity—or an individual’s psychological attachment to a rural area. Using data from the 2020 ANES, I find that rural identity predicts less favorable estimations of LGBT individuals. Rural identifiers are also less likely to support pro-LGBT policy measures than nonrural identifiers. Nevertheless, I find the magnitude of the effects of rural identity on anti-LGBT views to be surprisingly small. It is also the case that, on average, rural identifiers exhibit net-positive estimations of LGBT individuals and are broadly supportive of LGBT rights, suggesting that elected officials enacting anti-LGBT legislation in rural areas of the United States are potentially out of step with the preferences of their electorate. These findings also have implications for what it means to hold a rural identity beyond a generalized animosity toward urban areas, and for understanding urban-rural divergences in US public opinion on issues such as LGBT rights.

## Introduction

Attitudes toward individuals who identify as lesbian, gay, bisexual, or transgender (LGBT) have liberalized considerably in recent decades (Hicks and Lee 2006; Taylor et al. 2018; Lewis et al. 2022). Still, opposition to LGBT rights remains a fixture in contemporary American society. In June 2020, the US Supreme Court ruled in *Bostock v. Clayton County* that Title VII of the Civil Rights Act protects employees against discrimination because they are LGBT. Yet, since *Bostock*, local governments and state legislatures in predominately rural counties and states have proposed numerous anti-LGBT bills (FFaM 2022).

Anti-LGBT attitudes relate to intergroup attitudes and affect (Brewer 2003). In this paper, I draw on insights from social identity theory (Tajfel and Turner 1979; Tajfel 1981) to argue that an overlooked factor driving anti-LGBT attitudes is rural identity—or an individual’s psychological attachment to a rural area. Extant work on place-based identity conceptualizes individuals with rural identities as being affectively opposed to urban areas (Lyons and Utych 2021; Munis 2022). This is because individuals with rural identities perceive urban areas as looking down on rural areas, excluding them from decision making, and receiving a greater share of economic resources (Cramer 2016). Recent work expands on this argument, postulating that the term “urban” might be a blanket term for out-groups who tend to cluster within urban areas (Nelsen and Petsko 2021).

In this paper, I extend this argument by positing that a prominent “urban affiliated” group are individuals who identity as LGBT. LGBT individuals are not randomly distributed across different types of communities, but rather tend to cluster within urban areas of the United States (Badgett et al. 2019). Work on LGBT placemaking suggests that many of the more “urbane” ideals of Queer life are not applicable to suburban areas (Bain and Podmore 2021) or rural areas (Gray 2009). As Gray (2009) argues, an emphasis on “coming out” and being visible in public spaces cannot be directly adapted to nonurban contexts where LGBT individuals are less numerous, civic space is more limited, and community leaders are less tolerant. LGBT individuals are also likely to select into urban areas, which, on average, have a greater tolerance for diverse groups and have LGBT-friendly cultures (Weston 1995; Aldrich 2004; Annes and Redlin 2012).^1^

Place-based identities matter for understanding geographic differences in US public opinion. What qualifies as urban or rural in a given state or region is constructed by those who live in it.^2^ Urban and rural can therefore be conceptualized as relative categories. This point is why scholars emphasize the importance of asking for self-reported community type (i.e., whether an individual perceives that they live in an urban or rural area), as well as the extent to which an individual identities with their community, within surveys (see Munis 2022). Because what qualifies as urban or rural to an individual likely varies from place to place, objective measures of urbanicity such as the RUCA and RUCC schemes may not adequately capture urban-rural differences in public opinion.^3^ This is, of course, not to say that objective measures of urbanicity are obsolete. As Nemerever and Rogers (2021) argue, objective measures of urbanicity ought to be used in contexts where the researcher is interested in the influence of government programs that use those same classification schemes to assign varying levels of treatment. Instead, Nemerever and Rogers (2021) suggest that subjective measures of urbanicity should be used in behavioral work, especially when they demonstrate that subjective measures do not align with objective measures in their replication of Mummolo and Nall (2017). In sum, objective measures of urbanicity have their uses, but subjective measures of urbanicity may be better placed to understand urban-rural divergences in mass attitudes and behaviors.

Empirically, I draw on data from the 2020 ANES. Overall, I find that rural identity predicts less positive feelings toward LGBT individuals, as well as lower levels of support for LGBT rights. I also find that these effects vary based on the strength of an individual’s sense of rural belonging, where those with the strongest attachment to rural identity are less likely to support LGBT rights. Interestingly, though I find statistically significant effects through rural identity, the magnitude of these effects is rather small. Indeed, differences in affect toward LGBT individuals and support for LGBT rights when contrasting on rural identity are smaller relative to other factors such as party ID and religion. Moreover, though rural identifiers report less positive estimations of LGBT individuals and are less supportive of LGBT rights than nonrural identifiers, they still report net-positive estimations of LGBT individuals and are also broadly supportive of LGBT rights. These findings are noteworthy because they suggest that elected officials enacting anti-LGBT legislation in predominately rural areas (FFaM 2022) might be out of step with the preferences of their electorate.

This paper also contributes to a growing body of work attesting to the urban-rural gulf in US public opinion. Prior studies have used objective measures of place to test urban-rural differences in Americans’ political preferences (Scala and Johnson 2017; Albrecht 2019; Gimpel et al. 2020). However, recent work suggests that subjective measures of urbanicity may be especially relevant for understanding geographic polarization in political attitudes (Nemerever and Rogers 2021). This paper builds on this argument by highlighting how rural identity engenders greater opposition to LGBT rights, and how this relationship depends on an individual’s subjective identification with place.

## Public Opinion toward LGBT Individuals and Rights

There is an extensive body of work focusing on US public opinion toward individuals who identify as LGBT (Brewer 2003; Hicks and Lee 2006; Haider-Markel and Joslyn 2008). Still, much of the earlier work within the extant corpus focuses on attitudes toward lesbian, gay, and bisexual individuals as opposed to transgender individuals. Scholars find differences in levels of support for varying segments of the LGBT community. For instance, the public are more ambivalent about gay men than lesbians—differences that are likely reflective of societal gender stereotypes (Herek 2002). Still, more recent work suggests that gender stereotypes and attitudes toward gender conformity may also produce differences in attitudes toward other segments of the LGBT community—namely, toward transgender individuals (Becker and Jones 2021).

Studies on attitudes toward the transgender community indicate that the same demographic and psychological factors shaping attitudes toward LGB individuals—for instance, religiosity, authoritarianism, disgust sensitivity, and the importance of biological attributions—likewise shape attitudes toward transgender individuals (Miller et al. 2017; Castle 2019; Bowers and Whitley 2020). However, while attitudes toward transgender individuals are likely correlated with attitudes toward LGB individuals, work finds that attitudes toward transgender individuals are significantly more negative (Lewis et al. 2017),^4^ a fact that has driven recent research attesting to high levels of public polarization on the issue of transgender rights (Harrison and Michelson 2017). The divergence in US public opinion on transgender versus LGB rights has also motivated a number of experimental studies primarily interested in potential interventions that may work to reduce transphobic attitudes. For instance, research suggests that door-to-door canvassing may increase support for transgender nondiscrimination laws (Broockman and Kalla 2016). Elsewhere, Flores et al. (2021) report that perspective taking reduces transphobia among individuals who comply fully with such exercises—an important finding given the causal link between a reduction in transphobic attitudes and increased support for transgender rights (Flores et al. 2018a).

## Rural Identity and LGBT Attitudes

Social identity theory (SIT) posits that social identity occurs when a group member derives self-esteem from group membership. Individuals with a social identity adopt the various norms, interests, and values thought to be characteristic to the group, and feel a strong psychological attachment to the group and fellow group members (Tajfel and Turner 1979; Tajfel 1981). Social identification does not occur if one is simply part of a group, as this is group membership. For instance, a respondent might be considered a “rural American” if they reported living in a rural area on a survey, but residence alone may not be psychologically meaningful to the individual in question. In this way, social identification necessarily includes a psychological attachment to a given in-group identity. Because increased self-esteem or other psychological benefits influence social identification, group identifiers promote positive in-group distinctions (Tajfel and Turner 1979; Tajfel 1981).

There are different ways to promote position in-group distinctions. One way is to establish values associated with the group. This can be seen along place-based lines (Wuthnow 2018). The aim of attaching positive values to a group and its members is to promote the group’s status in society. Another way of creating a positive distinction is to give negative attributes to out-group members, especially those perceived as threatening to group interests. Doing so provides a contrast, thus making the in-group seem superior. One way of doing this is to engender a sense of “otherness” from those who are perceived as threatening to group interests. Urban areas are typically seen as the out-group within the extant political geography scholarship (Cramer 2016; Lyons and Utych 2021; Munis 2022). However, which groups qualify as urban affiliated may have important implications for what rural Americans view as out-groups. In recent work, for instance, Nelsen and Petsko (2021) find that rural Wisconsinites perceive the typical urban resident within the state to be a person of color (PoC). This is because individuals high in rural consciousness tend to hold racialized stereotypes of urban areas, perceiving cities with large non-White populations as “hubs for lazy, undeserving welfare recipients” (Nelsen and Petsko 2021).

Extant work finds rural identity to be a politically relevant construct. For instance, Lyons and Utych (2021) find that rural and urban areas are affectively polarized against one another. This affective dimension is important to the existence of rural identity because appeals to certain groups (i.e., rural Americans) hint to the existence of a group whose identity is derived from place. Elsewhere, Cramer (2016) argues that rural Wisconsinites feel looked down on and forgotten by those in Madison and Milwaukee, the two largest metro areas in the state. Urban areas are also perceived as sophisticated, progressive, and centers of creativity, whereas nonurban areas are characterized as backwards and ignorant (Lichter and Brown 2011).

I posit that another urban-affiliated out-group are LGBT individuals. Rural identifiers may exhibit anti-LGBT attitudes because of a number of core tenets underpinning their identities, including community solidarity (Hofferth and Iceland 2011) and anti-statist attitudes (Ashwood 2018). When individuals in rural areas come out as LGBT, the act itself is perceived as an affront to community sameness. Some cis/straight rural Americans perceive coming out and identifying as LGBT as “flaunting” and/or “forcing” a lifestyle on fellow community members (Boulden 2001). Accordingly, some LGBT individuals who live in rural areas “stay in the closet” and do not claim an openly Queer identity.

Beyond claiming an LGBT identity as a marker of difference, rural identifiers might also perceive that LGBT individuals violate the values that comprise community solidarity due to the urban connotations of LGBT individuals and rights. America’s largest cities have long served as imaginary and tangible places where individuals with noncisgender and nonheteronormative identities migrate from nonurban areas, “come out,” and form communities with similar individuals (Weston 1995; Aldrich 2004; Annes and Redlin 2012). The sheer size of some of the largest cities makes them likelier than rural areas to develop a “critical mass” of like-minded individuals (Tarrow 2022). The urban spaces within which LGBT individuals meet are important locales for Queer political mobilization within America’s largest cities. Much of our contemporary understanding for why LGBT individuals are a politically relevant demographic also comes from urban areas (Bailey 1999). This perspective suggests that the mechanism driving whether rural identifiers perceive LGBT individuals as urban is likely to be different to those that shape those of other groups—for instance, PoC.^5^

Though LGBT individuals have an affiliation with urban areas because of this history, it is not the case that all Queer individuals are alike when contrasting on community type. Because rural LGBT individuals tend to be less open with gender identity or sexuality than LGBT individuals residing in urban areas (Bell and Valentine 1995), rural identifiers are exposed to iterations of LGBT individuals via television media and computer-mediated communication (CMC). The media play an important role in promulgating the meaning of LGBT identity, which is widely received and understood by the mass public (Gross 2001). Networks have developed urban gay “stock” characters for their shows (Kazyak 2011). These characters come loaded with a variety of urban stereotypes. For instance, one conceptualization of gay “stock” characters is that they tend to be activists. To rural identifiers, however, activism can be a loaded term. This is because activism may suggest questioning well-established norms and calling attention to oneself (the antonym of community solidarity). Activism also conflicts with the rural-identity tenet of anti-statist attitudes (Ashwood 2018), since activism may entail asking for help from local, state, or federal governments.

While the in-group/out-group theoretical framework motivates why we would expect rural identifiers to have a low opinion of LGBT individuals, it is important to qualify that out-group animosity may not exhibit as strong of a relationship with other facets of LGBT public opinion, including the extent of support for pro-LGBT policies. Measures of LGBT group affect and LGBT policy support often correlate (see Brewer 2003), but this is not *always* the case. It is possible, for instance, for individuals to exhibit less favorable opinions toward a given group in society. However, this may not equate to believing that the group in question should be deprived of their civil rights.^6^ For these reasons, I propose two distinct hypotheses concerning the relationship between rural identity and LGBT attitudes:

**H1**: Individuals who identify as rural have lower estimations of LGBT individuals than nonrural identifiers.

**H2**: Individuals who identify as rural are less likely to support LGBT rights than nonrural identifiers.

Furthermore, identity alone seldom drives negative out-group affect and opposition to policies that benefit out-group members. For instance, Huddy (2001) identifies gradations in social identity strength, with identities having greater meaning to some individuals and less meaning to others. Additional work finds that stronger identification with and greater salience of social identities are more likely to engender the political effects of identity (Huddy et al. 2015). Consistent with these findings, Iyengar et al. (2019) find that the increased salience of partisan identity contributes to greater levels of affective polarization. I expect rural identity to function in a similar manner, whereby the effects of rural identity on feelings toward LGBT individuals and on levels of support for LGBT rights will be strongest for those with the highest levels of place-based identity salience:

**H3**: Individuals with stronger rural identities have lower estimations of LGBT individuals than individuals with weaker rural identities.

**H4**: Individuals with stronger rural identities are less likely to support LGBT rights than individuals with weaker rural identities.

## Data and Methods

### Data

To test these hypotheses, I draw on data from the 2020 ANES Time Series Study. The survey is a random probability-based, nationally representative mixed-mode study of phone interviews and online questionnaires (N = 8,280) (ANES 2022). The 2020 ANES is composed of a pre-election wave, with data collected from August 18, 2020, through to election day on November 3, 2020, as well as a postelection wave, with data collected from November 8, 2020, through to January 4, 2021. Because the place-based identity item was not included in the pre-election sample, I limit my analytical sample to ANES respondents who completed the postelection wave of the survey (N = 7,499). The overall response rate (RR) for reinterviews in the postelection wave of the 2020 ANES was 73.2 percent (ANES 2022).^7^ Missing data within the ANES dataset, including item nonresponse and “don’t know” responses, were handled via listwise deletion of cases. In addition, all descriptive and multivariate analyses to follow use the postelection weight provided by the ANES to ensure that inferences are generalizable to the US adult population.

### Dependent Measures

To measure group-based affect, the 2020 ANES contains two 101-point feeling thermometers that tap into respondents’ feelings toward gays and lesbians, as well as transgender individuals. A rating of 100 is indicative of “very warm or favorable feelings,” while a rating of 0 is indicative of “very cool or unfavorable feelings.”^8^ Given differences in support for gay and lesbian individuals versus transgender individuals (see Lewis et al. 2017; Jones et al. 2018), I estimate separate models for both thermometers.

The 2020 ANES also contains five items that gauge support for LGBT rights. The first item asks, “Do you think business owners who provide wedding-related services should be allowed to refuse services to same-sex couples if same-sex marriage violates their religious beliefs, or do you think business owners should be required to provide services regardless of a couple’s sexual orientation?” with possible responses ranging between 1 = “should be allowed” and 2 = “should not be allowed.” The second item asks, “Should transgender people—that is, people who identify themselves as the sex or gender different from the one they were born as—have to use the bathrooms of the gender they were born as, or should they be allowed to use the bathrooms of their identified gender?” with possible responses ranging between 1 = “have to use the bathrooms of the gender they were born as” and 2 = “be allowed to use the bathrooms of their identified gender.” The third item asks, “Do you favor or oppose laws to protect gays and lesbians against job discrimination?” with possible responses ranging between 1 = “favor” and 2 = “oppose.” The fourth item asks, “Do you think gay or lesbian couples should be legally permitted to adopt children?” with possible responses ranging between 1 = “yes” and 2 = “no.” The fifth and final item asks, “Which comes closest to your view?” with possible responses ranging between 1 = “gay and lesbian couples should be allowed to legally marry,” 2 = “gay and lesbian couples should be allowed to form civil unions but not legally marry,” and 3 = “there should be no legal recognition of gay or lesbian couples’ relationship.”^9^ Items one and two are reverse coded to indicate greater support for LGBT rights. I compile the five items into an overall index of LGBT rights (Cronbach’s α = 0.75).

### Rural Residence, Rural Identity, and Sense of Rural Belonging

My hypotheses imply that rural identifiers will have lower estimations of LGBT individuals and will be less likely to support LGBT rights even after accounting for an individual’s current place of residence. The logic behind this proposition is that individuals may identify as rural even though they do not live in a rural area.^10^ Moreover, recent work points to substantive variation in political values among rural residents who identify as rural versus rural residents who do not identify as rural (Trujillo 2022a). In this way, place-based measures of identity might still shape negative group-based affect and opposition to policy measures that benefit out-group members even after controlling for place. Because I am interested in quantifying the effects of rural identity on LGBT views *above and beyond* where a respondent lives, I therefore include a control for a respondent’s place of residence. Because respondent geocodes are not available in the 2020 ANES, I am unable to code an objective measure of urbanicity using administrative data. Therefore, I use responses to a survey question that asks respondents, “Do you currently live in a rural area, small town, suburb, or a city?” with possible responses ranging between 1 = “rural area,” 2 = “small town,” 3 = “suburb,” and 4 = “city.” My approach of using a subjective measure of urbanicity is consistent with that of Munis (2022), and also follows the recommendations of Nemerever and Rogers (2021), who suggest that objective measures of urbanicity are better utilized when researchers wish to understand the influence of government programs that use those objective classification schemes to assign varying levels of treatment.

The 2020 ANES also contains a categorical variable that asks, “Regardless of where you currently live, do you usually think of yourself as a city person, a suburb person, a small-town person, a country or rural person, or something else?” with possible responses ranging between 1 = “city person,” 2 = “suburb person,” 3 = “small-town person,” 4 = “rural person,” and 5 = “something else.” I code respondents as having a rural identity if they identified as a rural person (1 = “rural identity,” “0 = “non-rural identity”).^11^ This approach differs from that of Munis (2022), where respondents who described living in either a small town or a rural area are coded as rural identifiers. While rural areas and small towns are sometimes collapsed into a single measure within the extant political geography scholarship (Wuthnow 2018), work suggests that small towns are somewhat distinct from rural areas in terms of their demographic composition and their political values (Vidich and Bensman 1968). Contemporary work also points to small towns having somewhat friendlier LGBT cultures than rural areas—for instance, hosting more Queer spaces, such as gay bars (Mattson 2020). Therefore, I opt not to collapse rural and small-town identifiers into a single measure of rural identity.^12^

**Figure 1.**
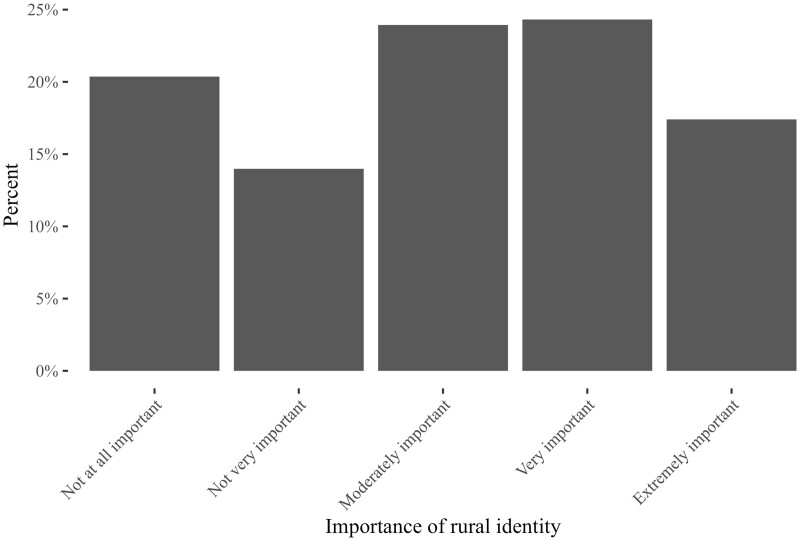
Distribution of respondents across different strengths of sense of rural belonging. Limited to respondents who identify as rural.

For sense of rural belonging, the 2020 ANES includes a five-point ordinal item that asks, “How important is being a rural person to your identity?” with possible responses ranging between 1 = “not at all important,” 2 = “a little important,” 3 = “moderately important,” 4 =  “very important,” and 5 = “extremely important.”^13^ For the interested reader, figure 1 graphs the distribution of respondents across different strengths of rural belonging.

### Covariates

Work indicates that anti-statist attitudes are pervasive in rural America (Ashwood 2018). Anti-statist attitudes may shape opposition to LGBT rights because individuals are opposed to government action.^14^ To test this possibility, I control for anti-statist attitudes. To measure anti-statist attitudes, I rely on a six-point ordinal item that asks respondents about their level of agreement with the statement “the less government, the better,” with possible responses ranging between 1 = “feels strongly that less government the better” and 6 = “feels strongly more things that government should be doing.”^15^

Models also include a number of demographic and structural covariates. Models control for contact with LGB individuals (1 = “LGB family member, friend, or coworker,” 0 = “no LGB family member, friend, or coworker”), party ID (7-point ordinal item ranging between 1 = “strong Democrat” and 7 = “strong Republican”), ideology (7-point ordinal item ranging between 1 = “extremely liberal” and 7 = “extremely conservative”), race (1 = “White,” 0 = “non-White”), age (in years), sex (1 = “female,” 0 = “male”), LGB identity (1 = “LGB,” 0 = “straight”), education (5-point ordinal item ranging between 1 = “less than high school credential” and 5 = “graduate degree”), family income (22-point ordinal variable ranging between 1 = “less than $5,000” and 22 = “$250,000 or more”), religion (1 = “Evangelical Protestant,” 0 = “non-Evangelical Protestant”), frequency of religious service attendance (5-point ordinal variable ranging between 1 = “never” and 5 = “every week”), and region (dichotomous items for “Midwest,” “South,” and “West,” with “Northeast” serving as the base category).

## Results

### Group-Based Affect and Support for LGBT Rights

**Figure 2.**
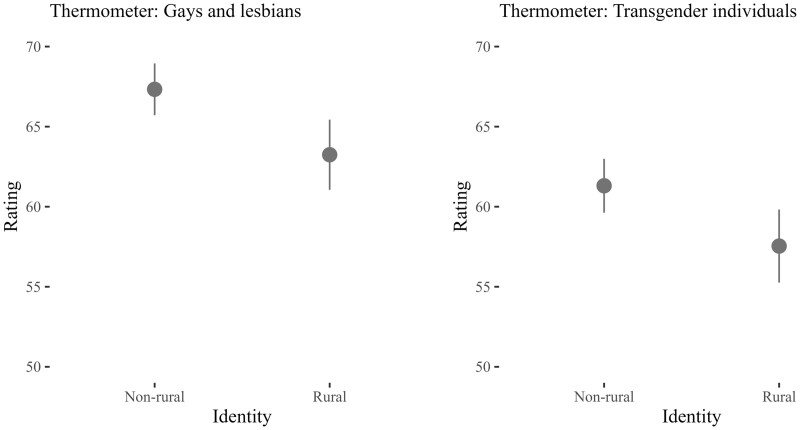
Rural identity and group-based affect. Points represent the predicted feeling thermometer ratings for gays and lesbians/transgender individuals by rural/nonrural identity. The vertical lines are 95 percent confidence intervals. Predicted probabilities calculated by holding all other variables in model constant or at their respective mean values. Full model estimates presented in Supplementary Material section D.

I begin by testing the relationship between rural identity and group-based affect. These models also control for subjective place of residence and my baseline set of covariates. The left panel in figure 2 graphs the predicted ratings of gays and lesbians by rural/nonrural identity. As indicated here, ANES respondents who do not identify as rural give gay and lesbian individuals a predicted rating of 67.33 points. By contrast, respondents who identify as rural give gays and lesbians a predicted rating of 63.24 points. This difference in group-based affect between rural identifiers is somewhat small at 4.09 points, but is nonetheless statistically significant (*p* = .001). Interestingly, the estimates for rural identity differ from whether respondents perceive that they live in a city or a rural area. ANES respondents who described living in a city give gay and lesbian individuals a predicted rating of 67.33 points. Conversely, respondents who described their area as rural give gay and lesbian individuals a predicted thermometer rating of 64.43 points. Thus, the difference in affect toward gays and lesbians depending on whether individuals describe living in a city or rural area is 2.90 points—a somewhat smaller difference relative to when we contrast on rural identity.

Turning to the right panel in figure 2, we see that respondents who do not identify as rural give transgender individuals a predicted rating of 61.30 points. Conversely, respondents who identify as rural give transgender individuals a predicted rating of 57.54 points. This difference in group-based affect is, once again, relatively small at just 3.76 points, but is still a statistically significant contrast (*p* = .001). Again, how then might these estimates differ relative to whether respondents describe living in a city or a rural area? ANES respondents who described living in a city give transgender individuals a predicted thermometer rating of 61.30 points. By contrast, respondents who described their area as rural give transgender individuals a predicted thermometer rating of 60.29 points. Therefore, the difference in affect toward transgender individuals depending on whether individuals describe their area as a city or as rural is just 1.01 points. Once again, this is a smaller difference in affect relative to when we contrast on rural identity.

Overall, figure 2 suggests that rural identifiers have lower estimations of LGBT individuals than nonrural identifiers. Therefore, we find evidence in favor of **H1**. In contextualizing these results, however, it is important to note that rural identifiers provide ratings greater than 50 on both thermometer scales. This indicates that, on average, rural identifiers have somewhat favorable estimations of both groups. Furthermore, differences in group-based affect when contrasting on rural identity are smaller relative to other factors such as party ID and religion. For instance, the expected difference in levels of group-based affect between strong Democrats and strong Republicans is 8.23 points when it comes to estimations of gays and lesbians, and 10.20 points when it comes to estimations of transgender individuals. Similarly, the expected difference in levels of group-based affect between Evangelical Protestants and non-Evangelical Protestants is 7.28 points when it comes to estimations of gays and lesbians, and 5.28 points when it comes to estimations of transgender individuals.

**Figure 3.**
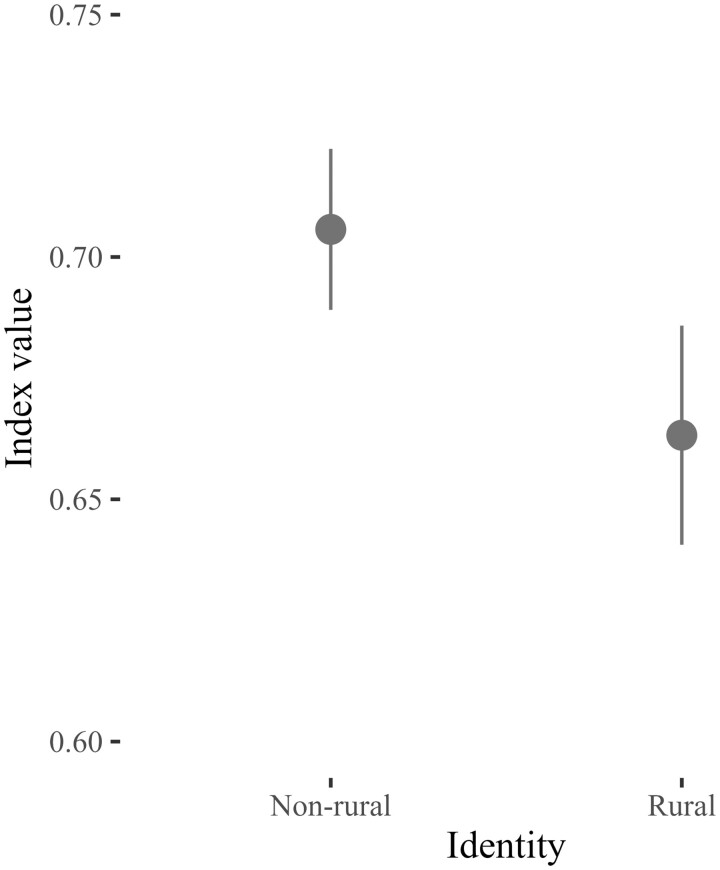
Rural identity and support for LGBT rights. Points represent the predicted level of support for LGBT rights by rural/nonrural identity. The vertical lines are 95 percent confidence intervals. Predicted probabilities calculated by holding all other variables in model constant or at their respective mean values. Full model estimates presented in Supplementary Material section E.

Next, figure 3 graphs the relationship between rural identity and support for LGBT rights. As indicated here, there is a statistically significant gap of 5.00 points (*p* = .001) in support for LGBT rights among rural and nonrural identifiers. On a normalized scale ranging between 0 and 1, the predicted level of support for LGBT rights among nonrural identifiers is 0.71. By contrast, the predicted level of support for LGBT rights among rural identifiers is 0.66. As was the case with the models for group-based affect, the effects of subjective urbanicity (i.e., whether respondents describe living in a city or a rural area) are of a lower magnitude relative to rural identity. Among ANES respondents who described living in a city, the predicted level of support for LGBT rights on the normalized scale is 0.71. Conversely, the predicted level of support for LGBT rights among respondents who described their area as rural is 0.70 on the normalized scale. Thus, the gap in support for LGBT rights when contrasting on respondents’ subjective evaluations of place is just 0.10 points, relative to 5.00 points for rural identity.

Overall, then, figure 3 supports the hypothesis that rural identifiers are less likely to support LGBT rights **(H2)**. However, as was the case with the models for group-based affect, the magnitude of the differences in support for LGBT rights when contrasting on rural/nonrural identity is rather small. Moreover, rural identifiers are above the midpoint of the index, suggesting that, on average, they are still somewhat supportive of LGBT rights.

My analyses thus far have relied on an overall index compiled from several items. However, it is possible that differences in support for LGBT rights among rural and nonrural identifiers are stronger for some measures (see Lewis et al. 2017). To test this possibility, I estimate separate models for each item used to compile the LGBT index variable. Overall, I find that rural identity does not predict whether individuals think businesses should be allowed to refuse service to same-sex couples if they believe that same-sex marriage violates their religious beliefs. However, rural identity *does* predict lower support for the remaining LGBT policy items. Therefore, I do not find consistency across models. Still, rural identity predicts lower support for 80 percent of the LGBT policy preferences measured in the 2020 ANES (full estimates presented in Supplementary Material section F).

### Rural Identity Moderated by Sense of Rural Belonging

**Figure 4.**
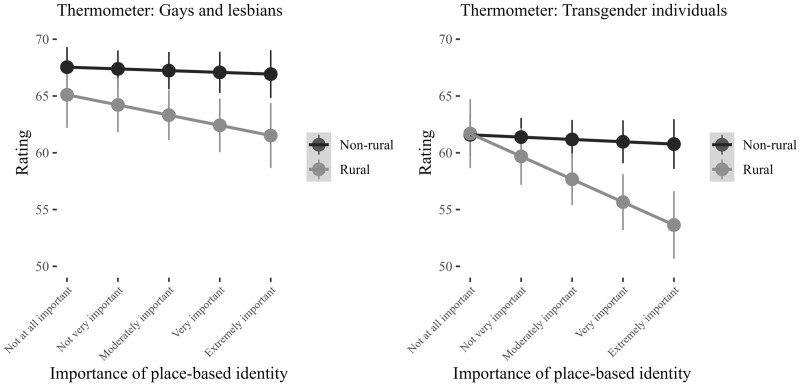
Rural identity and group-based affect, moderated by sense of rural belonging. Points represent the predicted feeling thermometer ratings for gays and lesbians/transgender individuals by rural/nonrural identity at each level of the strength of rural belonging scale. The vertical lines are 95 percent confidence intervals. Predicted probabilities calculated by holding all other variables in model constant or at their respective mean values. Full model estimates presented in Supplementary Material section G.

**H3** predicted that the effects of rural identity would be stronger for those with a greater sense of rural belonging. To test this expectation, I ran a series of additional models that included interaction terms between rural identity and strength of rural belonging. Figure 4 graphs the predicted thermometer ratings among rural and nonrural identifiers across levels of the sense of rural belonging measure. First, the interaction term for rural identity and sense of rural belonging is not significant in the model predicting feelings toward gays and lesbians. This is evidenced in the left panel in figure 4, where we observe minimal variance in favorability toward gays and lesbians among rural identifiers across levels of the sense of rural belonging measure.

When it comes to the transgender feeling thermometer, the interaction term between rural identity and sense of rural belonging is statistically significant (*p* = .001). As indicated by the right panel in figure 4, the effect is such that, as a respondent’s strength of rural belonging increases, rural identifiers are expected to exhibit less positive feelings toward transgender individuals. A respondent who sees themselves as a rural person but who considers their rural identity to be “not at all important” gives transgender individuals a thermometer rating of 61.69. By contrast, a respondent who sees themselves as a rural person and who considers this identity to be “extremely important” gives transgender individuals a thermometer rating of 53.64. Therefore, increases in the sense of rural belonging are associated with an 8.05-point decrease in favorable estimations of transgender individuals among rural identifiers.

**Figure 5.**
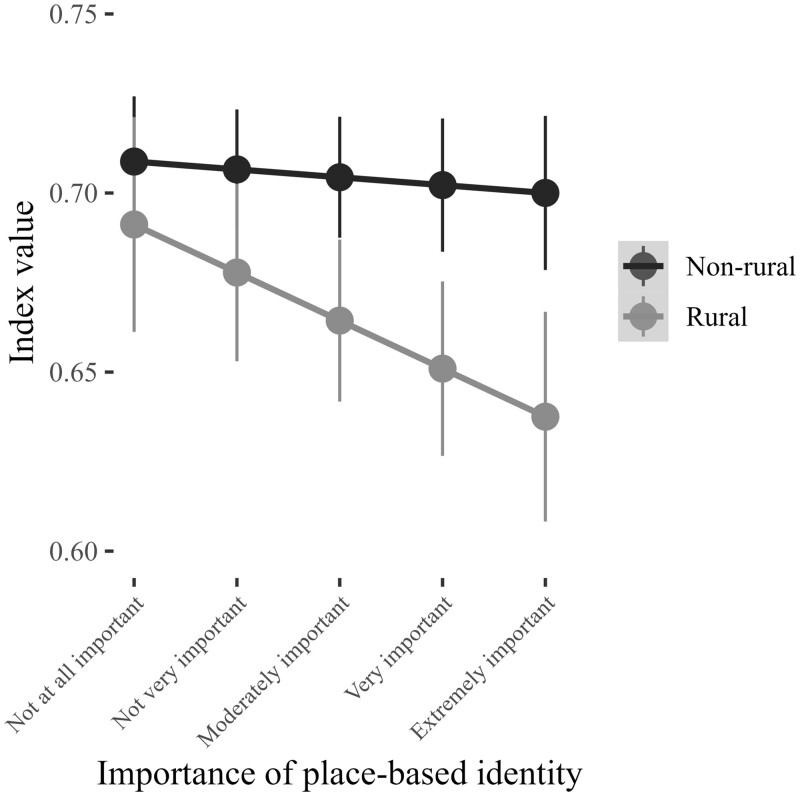
Rural identity and support for LGBT rights, moderated by sense of rural belonging. Points represent the predicted level of support for LGBT rights by rural/nonrural identity at each level of the sense of rural belonging measure. The vertical lines are 95 percent confidence intervals. Predicted probabilities calculated by holding all other variables in model constant or at their respective mean values. Full model estimates presented in Supplementary Material section H.

Finally, figure 5 graphs the expected level of support for LGBT rights among rural/nonrural identifiers across levels of the sense of rural belonging measure. In the model, the interaction term between rural identity and sense of rural belonging is statistically significant (*p* = .010). The nature of the interaction can be seen more clearly below in figure 5. The effect is such that, as an individual’s sense of rural belonging increases, individuals who identify as rural are less likely to support LGBT rights. A respondent who sees themselves as a rural person but who considers their rural identity to be “not at all important” scores 0.69 on the LGBT rights index. Conversely, a respondent who sees themselves as a rural person and who considers their rural identity to be “extremely important” scores 0.64 on the LGBT rights index. As such, increases in one’s sense of rural belonging are associated with a 5.00-point decrease in the predicted probability that a rural identifier will support LGBT rights.

## Conclusion

Overall, I find consistent evidence in favor of my hypotheses. Rural identifiers view LGBT individuals somewhat less positively and are somewhat less supportive of LGBT rights than those who do not identify as rural **(H1–H2)**. This relationship is moderated by the strength of an individual’s sense of rural belonging, whereby those who identify more strongly as rural have cooler estimations of LGBT individuals and are the least supportive of LGBT rights **(H3–H4)**.

The results have three important implications. First, given the ongoing passage of numerous anti-LGBT pieces of legislation in predominantly rural counties and states (FFaM 2022), we might have expected to observe larger or more consequential effects through rural identity on the measures of group affect and LGBT rights support. However, I find the magnitude of the effects of rural identity on LGBT attitudes to be somewhat small. It is likewise the case that rural identifiers exhibit net-positive estimations of LGBT individuals, and are also broadly supportive of LGBT rights (albeit at marginally lower rates than nonrural identifiers). The findings are important in this context because they point to an interesting disconnect between rural identifiers’ attitudes toward LGBT individuals on the one hand, and the actions of some county and state officials to pass anti-LGBT legislation on the other. Thus, one takeaway is that elected officials enacting anti-LGBT legislation in rural areas throughout the United States are potentially out of step with the preferences of their electorate.

Second, the findings emphasize that political scientists should think about what exactly “rural” means when they want to measure the impact of place on political attitudes and behaviors. Recent work underscores this point with respect to objective measures of rurality. For instance, Nemerever and Rogers (2021) suggest that researchers should defer to objective schema when one is primarily interested in the influence of government programs that use those same classification schemes. However, what qualifies as “rural” to an individual may differ to what the Economic Research Service (ERS) defines as a nonmetro area. There is also an affective dimension to the urban-rural divide, whereby those who see themselves as rural are opposed to those in urban areas of the United States (Lyons and Utych 2021). For these reasons, researchers might be better placed gauging individuals’ subjective conceptualizations of place (as is the focus of this paper), as well as the extent of their psychological attachment to place (Munis 2022). Recent work demonstrates the relevance of place-based identities for understanding rural-urban differences in a variety of political attitudes and behaviors (Lyons and Utych 2021; Trujillo 2022b), and that this psychological attachment to rurality is not necessarily tied to whether individuals live in a rural area (Trujillo 2022a). In many cases here, I find the size of the coefficients for rural identity are larger than those for subjective urbanicity, suggesting that, when it comes to understanding public opinion on LGBT individuals and rights, the extent to which individuals see themselves as rural matters more than whether they actually describe living in a rural area. Researchers accounting for urban-rural effects in studies of LGBT public opinion should therefore include a control for place-based identity where available, as I find that this measure is more closely related to my outcomes.

Finally, the results build on extant work concerning rural identifiers’ attitudes toward various groups in US society. Recent work argues that the term “urban” may function as a blanket term for out-groups, including PoC (Nelsen and Petsko 2021). I have argued that LGBT individuals might be conceptualized as an “urban-affiliated” group given the clustering of LGBT populations within urban areas of the United States (Badgett et al. 2019), as well as how visible LGBT culture and Queer political mobilization emerged from America’s largest cities (Bailey 1999). By demonstrating that rural identifiers have somewhat less favorable estimations of LGBT individuals and are somewhat less supportive of LGBT rights, my findings indicate that rural identity is also associated with negative attitudes toward those with concealable stigmatized identities^16^—for instance, LGBT individuals (Herek and Capitanio 1996).

## Supplementary Material

nfad045_Supplementary_Data

## Data Availability

Replication data and documentation are available at https://doi.org/10.7910/DVN/6LZREZ.

## Endnotes

1 Still, work finds that exposure to different groups via resource competition (Esses et al. 2002), changing population demographics (Evans and Ivaldi 2021), and in segregated environments (Enos 2017) may engender prejudice.

2 Places like Cheyenne (pop 65,051), and Laramie (pop 31,359) are large cities in the context of Wyoming, the least populous state, and residents of Wyoming likely understand them as such. However, such places might not be considered large cities to residents of California, the most populous state. Indeed, if Cheyenne and Laramie were suddenly annexed by California, they would be the 141st and 259th largest cities within the nation’s most populous state, respectively.

3 Rural-urban commuting area (RUCA) codes classify US census tracts using measures of population density, urbanization, and daily commuting, whereas Rural-Urban Continuum Codes (RUCC) are an urban-rural classification scheme that distinguishes metropolitan counties by the population size of their metro area, and nonmetropolitan counties by degree of urbanization and adjacency to a metro area.

4 One factor that might explain these differences is interpersonal contact. Contact with sexual minorities reduces homophobia (Herek and Capitanio 1996). However, Flores (2015) found that contact with transgender individuals did not have a significant effect in reducing prejudice. Still, recent studies suggest that contact with transgender individuals may actually reduce transphobic attitudes (Tadlock et al. 2017). In addition to interpersonal contact, research indicates that mere exposure effects with transgender individuals via computer-mediated communication (CMC) may reduce transphobia (Flores et al. 2018b).

5 It is important to qualify that the data I use to test my hypotheses lack specific measures to test whether rural identifiers actually perceive LGBT individuals as urban affiliated.

6 Extant work in European context likewise points to a divergence between how individuals may feel toward LGBT persons on the one hand, and whether they are likely to support LGBT rights on the other (Dotti Sani and Quaranta 2020).

7 This response rate is based on AAPOR’s response rate calculation Response Rate 1 (RR1). This estimate is the number of complete interviews divided by the number of interviews (complete plus partial) plus the number of noninterviews (refusal and break-off plus noncontacts plus others) plus all cases of unknown eligibility (AAPOR 2016).

8 Full wordings for all items are presented in Supplementary Material section A.

9 This item is dichotomized to allow for inclusion into the overall index of LGBT rights support.

10 Descriptive analyses of the ANES data reveal that, among respondents who identify as rural, 58.67 percent describe living in a rural area. By contrast, 8.28 percent of rural identifiers describe living in a city, 7.20 percent describe living in a suburb, while 21.21 percent describe living in a small town (weighed estimates). These statistics provide some indication that respondents are willing to adopt a rural identity irrespective of whether or not they describe hailing from a rural part of the United States.

11 Given that some variability may be lost with a dichotomous measure, I also model rural identity using the untransformed ordinal item. Results are substantively similar to those using the dichotomized item in the main paper and are presented in Supplementary Material section B.

12 As a robustness test, I assess whether the results are an artefact of my decision not to collapse “small town” and “rural” identifiers into a single measure of rural identity. I do this by estimating a series of additional models for group-based affect and support for LGBT rights where the key variable of interest is a collapsed measure of rural/small-town identity. Results are presented in Supplementary Material section C and are substantively similar to those presented in the main paper.

13 I refer to this measure as “sense of rural belonging” because it does not include components that capture a sense of shared fate with fellow rural identifiers, or perceptions of discrimination against rural identifiers—components that are important because they contribute to the salience of group identity (see Miller et al. 1981).

14 In the case of LGBT rights, this might include the legalization of same-sex marriage, or the passage of laws that prohibit discrimination against LGBT individuals in employment.

15 The item is reverse coded so that higher values indicate more salient anti-statist attitudes.

16 Concealable stigmatized identities are those which can be kept hidden from others, but which also have negative stereotypes attached that may still result in discrimination.
