# Supplementary material for: Rural Identity and LGBT Public Opinion in the United States
Source: Public Opin Q. 2023 Nov 3;87(4):956–77. doi: 10.1093/poq/nfad045 (PMC10748466; doi:10.1093/poq/nfad045)
Supplement: nfad045_Supplementary_Data [file nfad045_supplementary_data.pdf]

Rural identity and LGBT public opinion in the United States

Jack Thompson

University of Exeter

Supplementary Material

A Item Wording **2**

B Place Identity Modelled as an Ordinal Measure **16**
B.1 Group-Based Affect 16
B.2 Support for LGBT rights 17
B.3 Rural Identity Moderated by Sense of Rural Belonging 18

C Collapsing "Rural" and "Small-Town" into a Single Measure of Rural Identity **20**
C.1 Group-Based Affect 20
C.2 Support for LGBT rights 21
C.3 Rural/Small-Town Identity Moderated by Sense of Rural Belonging 22

D Testing the Relationship Between Rural Identity and Group-Based Affect **24**

E Testing the Relationship Between Rural Identity and Support for LGBT Rights **25**

F Testing the Relationship Between Rural Identity and Support for LGBTQ Rights (Individual Items) **26**

G Testing for Moderation Between Rural ID and Sense of Rural Belonging (Group-Based Affect) **27**

H Testing for Moderation Between Rural ID and Sense of Rural Belonging (Support for LGBTQ Rights) **28**

A Item Wording

Feeling thermometers

Gay men and lesbians

We'd like to get your feelings toward some of our political leaders and other people who are in the news these days. I'll read the name of a person and I'd like you to rate that person using something we call the feeling thermometer.

Ratings between 50 degrees and 100 degrees mean that you feel favorable and warm toward the person. Ratings between 0 degrees and 50 degrees mean that you don't feel favorable toward the person and that you don't care too much for that person. You would rate the person at the 50 degree mark if you don't feel particularly warm or cold toward the person.

How would you rate: Gay men and lesbians

-9. Refused
-7. No post-election data, deleted due to incomplete interview
-6. No post-election interview
-5. Interview breakoff
-4. Technical error
0
1
2
3
4
5
6
7
8
9
10
15
16

20
25
30
35
38
40
45
48
49
50
51
52
54
55
57
59
60
65
69
70
75
76
77
78
79
80
82
83
85
86
87
88
89
90
95
98

99
100
998. Don’t know

Transgender individuals

We’d like to get your feelings toward some of our political leaders and other people who are in the news these days. I’ll read the name of a person and I’d like you to rate that person using something we call the feeling thermometer.

Ratings between 50 degrees and 100 degrees mean that you feel favorable and warm toward the person. Ratings between 0 degrees and 50 degrees mean that you don’t feel favorable toward the person and that you don’t care too much for that person. You would rate the person at the 50 degree mark if you don’t feel particularly warm or cold toward the person.

How would you rate: Transgender people

9. Refused
-7. No post-election data, deleted due to incomplete interview
-6. No post-election interview
-5. Interview breakoff
-4. Technical error
0
1
2
3
4
5
8
10
12
13
15
20
25

27
30
35
40
42
44
45
48
49
50
51
52
55
60
61
63
65
66
67
68
70
72
75
76
77
78
79
80
84
85
86
89
90
95
96
98

99
100
998. Don’t know

LGBT rights items

Services to same-sex couples
Do you think business owners who provide wedding-related services should be allowed to refuse services to same-sex couples if same-sex marriage violates their religious beliefs, or do you think business owners should be required to provide services regardless of a couple’s sexual orientation?

9. Refused
-8. Don’t know
1. Should be allowed to refuse
2. Should be required to provide services

Transgender policy
Should transgender people – that is, people who identify themselves as the sex or gender different from the one they were born as – have to use the bathrooms of the gender they were born as, or should they be allowed to use the bathrooms of their identified gender?

-9. Refused
-8. Don’t know
1. Have to use the bathrooms of the gender they were born as
2. Be allowed to use the bathrooms of their identified gender

Should laws protect gays and lesbians against job discrimination
Do you favor or oppose laws to protect gays and lesbians against job discrimination?

-9. Refused
-8. Don’t know
1. Favor
2. Oppose

Should gay and lesbian couple be allowed to adopt

Do you think gay or lesbian couples should be legally permitted to adopt children?

-9. Refused
-8. Don't know
1. Yes
2. No

R position on same-sex marriage
Which comes closest to your view? You can just tell me the number of your choice.

9. Refused
-8. Don't know
1. Gay and lesbian couples should be allowed to legally marry
2. Gay and lesbian couples should be allowed to form civil unions but not legally marry
3. There should be no legal recognition of gay or lesbian couples' relationship

Does R currently live in an urban or rural area

Do you currently live in a rural area, small town, suburb, or a city?

-9. Refused
-8. Don't know
-7. No post-election data, deleted due to incomplete interview
-6. No post-election interview
-5. Interview breakoff
1. Rural area
2. Small town
3. Suburb
4. City

Does R usually think of self as rural or urban person

Regardless of where you currently live, do you usually think of yourself as a city person, a suburb person, a small-town person, a country or rural person, or something else?

9. Refused
-8. Don’t know
-7. No post-election data, deleted due to incomplete interview
-6. No post-election interview
-5. Interview breakoff
1. City person
2. Suburb person
3. Small-town person
4. Country (or rural) person
5. Something else SPECIFY

How important is rural or urban to R’s identity

How important is being a [city person / suburb person / small-town person / country or rural person] to your identity?

[Not at all important, a little important, moderately important, very important, or extremely important / Extremely important, very important, moderately important, a little important, or not at all important]?

9. Refused
-8. Don’t know
-7. No post-election data, deleted due to incomplete interview
-6. No post-election interview
-5. Interview breakoff
-1. Inapplicable
1. Not at all important
2. A little important
3. Moderately important
4. Very important
5. Extremely important

SUMMARY: Less or more government

-7. No post-election data, deleted due to incomplete interview
-6. No post-election interview

-5. Interview breakoff
-2. DK/RF
1. Feels strongly less government better
2. Feels somewhat strongly less government better
3. Feels not strongly less government better
4. Feels not strongly more things government should be doing
5. Feels somewhat strongly more things government should be doing
6. Feels strongly more things government should be doing

Sexual orientation of family and friends
Among your immediate family members, relatives, neighbors, co-workers, or close friends, are any of them gay, lesbian, or bisexual as far as you know?

-7. No post-election data, deleted due to incomplete interview
-6. No post-election interview
-5. Interview breakoff
1. Yes
2. No

SUMMARY: Party ID

9. Refused
-8. Don't know
1. Strong Democrat
2. Not very strong Democrat
3. Independent-Democrat
4. Independent
5. Independent-Republican
6. Not very strong Republican
7. Strong Republican

7pt scale liberal conservative self-placement
Where would you place yourself on this scale, or haven't you thought much about this?

-9. Refused
-8. Don't know

1. Extremely liberal
2. Liberal
3. Slightly liberal
4. Moderate; middle of the road
5. Slightly conservative
6. Conservative
7. Extremely conservative
99. Haven't thought much about this

SUMMARY: R self-identified race/ethnicity

9. Refused
-8. Don't know
1. White, non-Hispanic
2. Black, non-Hispanic
3. Hispanic
4. Asian or Native Hawaiian/other Pacific Islander, non-Hispanic alone
5. Native American/Alaska Native or other race, non-Hispanic alone
6. Multiple races, non-Hispanic

SUMMARY: Respondent age

9. Refused
18
19
20
21
22
23
24
25
26
27
28
29
30

67
68
69
70
71
72
73
74
75
76
77
78
79
80. Age 80 or older

What is your (R) sex

What is your sex?

-9. Refused
1. Male
2. Female

Sexual orientation of R

Do you consider yourself to be heterosexual or straight, homosexual or gay, or bisexual?

-9. Refused
-5. Interview breakoff (sufficient partial IW)
1. Heterosexual or straight
2. Homosexual or gay (or lesbian)
3. Bisexual
4. Something else SPECIFY

SUMMARY: Respondent 5 Category level of education

9. Refused
-8. Don’t know
-2. Missing,
1. Less than high school credential
2. High school credential
3. Some post-high school, no bachelor’s degree
4. Bachelor’s degree
5. Graduate degree

SUMMARY: Total (family) income

-9. Refused
-5. Interview breakoff
1. Under $9,999
2. $10,000-14,999
3. $15,000-19,999
4. $20,000-24,999
5. $25,000-29,999
6. $30,000-34,999
7. $35,000-39,999
8. $40,000-44,999
9. $45,000-49,999
10. $50,000-59,999
11. $60,000-64,999
12. $65,000-69,999
13. $70,000-74,999
14. $75,000-79,999
15. $80,000-89,999
16. $90,000-99,999
17. $100,000-109,999
18. $110,000-124,999
19. $125,000-149,999
20. $150,000-174,999
21. $175,000-249,999
22. $250,000 or more

What is present religion of R

What is your present religion, if any?

-9. Refused
-8. Don't know
1. Protestant
2. Roman Catholic
3. Orthodox Christian (such as Greek or Russian Orthodox)
4. Latter-Day Saints (LDS)
5. Jewish
6. Muslim
7. Buddhist
8. Hindu
9. Atheist
10. Agnostic
11. Something else
12. Nothing in particular

Does Christian consider self born-again

Would you call yourself a born-again Christian, that is, have you personally had a conversion experience related to Jesus Christ?

-9. Refused
-1. Inapplicable
1. Yes
2. No

Attend religious services how often

Do you go to religious services every week, almost every week, once or twice a month, a few times a year, or never?

-9. Refused

-8. Don’t know
-1. Inapplicable
1. Every week
2. Almost every week
3. Once or twice a month
4. A few times a year
5. Never

Census region

1. Northeast
2. Midwest
3. South
4. West

B Place Identity Modelled as an Ordinal Measure

B.1 Group-Based Affect

OLS Estimates of Relationship Between Place Identity and Group-Based Affect

	Gays and lesbians	Transgender individuals
(Intercept)	80.988	81.747
	SE = 1.716	SE = 1.782
	p = 0.000	p = 0.000
Place identity	−1.553	−1.639
	SE = 0.322	SE = 0.334
	p = 0.000	p = 0.000
Suburb	0.018	−0.361
	SE = 0.762	SE = 0.791
	p = 0.981	p = 0.648
Small town	1.019	1.832
	SE = 0.872	SE = 0.905
	p = 0.243	p = 0.043
Rural area	−2.522	−3.881
	SE = 1.024	SE = 1.065
	p = 0.014	p = 0.000
Anti-state attitudes	−0.025	−0.494
	SE = 0.175	SE = 0.182
	p = 0.886	p = 0.007
Contact with LGBT individuals	12.002	10.161
	SE = 0.612	SE = 0.636
	p = 0.000	p = 0.000
Party ID	−1.354	−1.711
	SE = 0.188	SE = 0.196
	p = 0.000	p = 0.000
Ideology (Conservative)	−3.073	−3.706
	SE = 0.286	SE = 0.298
	p = 0.000	p = 0.000
White	2.904	1.490
	SE = 0.688	SE = 0.714
	p = 0.000	p = 0.037
Age	−0.127	−0.105
	SE = 0.017	SE = 0.018
	p = 0.000	p = 0.000
Female	5.784	5.772
	SE = 0.580	SE = 0.603
	p = 0.000	p = 0.000
LGB	7.321	6.764
	SE = 1.172	SE = 1.219
	p = 0.000	p = 0.000
Education	1.053	1.155
	SE = 0.276	SE = 0.287
	p = 0.000	p = 0.000
Family income	0.188	0.130
	SE = 0.050	SE = 0.051
	p = 0.000	p = 0.011
Evangelical Protestant	−7.350	−5.306
	SE = 0.804	SE = 0.831
	p = 0.000	p = 0.000
Church attendance	−2.355	−2.271
	SE = 0.206	SE = 0.214
	p = 0.000	p = 0.000
Midwest	−1.106	0.226
	SE = 0.918	SE = 0.956
	p = 0.229	p = 0.813
South	−1.170	−1.260
	SE = 0.843	SE = 0.876
	p = 0.165	p = 0.150
West	0.479	0.322
	SE = 0.911	SE = 0.947
	p = 0.599	p = 0.734
Num.Obs.	6472	6459
R2	0.295	0.302
R2 Adj.	0.293	0.300
AIC	61 403.1	61 762.9
BIC	61 545.4	61 905.1
Log.Lik.	−30 680.539	−30 860.438
F	142.215	146.461
RMSE	22.87	23.75

B.2 Support for LGBT rights

OLS Estimates of Relationship Between Place Identity and Support for LGBT Rights

	Model 1
(Intercept)	1.039<br>SE = 0.018<br>p = 0.000
Place identity	−0.016<br>SE = 0.003<br>p = 0.000
Suburb	0.019<br>SE = 0.008<br>p = 0.018
Small town	0.020<br>SE = 0.009<br>p = 0.028
Rural area	−0.005<br>SE = 0.011<br>p = 0.609
Anti-state attitudes	−0.014<br>SE = 0.002<br>p = 0.000
Contact with LGBT individuals	0.084<br>SE = 0.006<br>p = 0.000
Party ID	−0.024<br>SE = 0.002<br>p = 0.000
Ideology (Conservative)	−0.054<br>SE = 0.003<br>p = 0.000
White	0.042<br>SE = 0.007<br>p = 0.000
Age	−0.001<br>SE = 0.000<br>p = 0.000
Female	0.033<br>SE = 0.006<br>p = 0.000
LGB	0.040<br>SE = 0.012<br>p = 0.001
Education	0.011<br>SE = 0.003<br>p = 0.000
Family income	0.003<br>SE = 0.001<br>p = 0.000
Evangelical Protestant	−0.098<br>SE = 0.008<br>p = 0.000
Church attendance	−0.036<br>SE = 0.002<br>p = 0.000
Midwest	−0.027<br>SE = 0.009<br>p = 0.004
South	−0.033<br>SE = 0.009<br>p = 0.000
West	−0.026<br>SE = 0.009<br>p = 0.005
Num.Obs.	6311
R2	0.429
R2 Adj.	0.427
AIC	1897.9
BIC	2039.7
Log.Lik.	−927.961
F	248.443
RMSE	0.23

B.3 Rural Identity Moderated by Sense of Rural Belonging

OLS Estimates of Relationship Between Place Identity and Group-Based Affect (Interactive Effects for Sense of Rural Belonging)

	Gays and lesbians	Transgender individuals
(Intercept)	80.323	79.587
	SE = 2.198	SE = 2.283
	p = 0.000	p = 0.000
Place identity	−0.822	0.015
	SE = 0.603	SE = 0.626
	p = 0.172	p = 0.982
Sense of rural belonging	0.227	0.779
	SE = 0.533	SE = 0.553
	p = 0.670	p = 0.159
Place identity × sense of rural belonging	−0.249	−0.585
	SE = 0.198	SE = 0.206
Suburb	−0.117	−0.595
	SE = 0.766	SE = 0.794
	p = 0.879	p = 0.454
Small town	0.943	1.673
	SE = 0.873	SE = 0.905
	p = 0.280	p = 0.065
Rural area	−2.421	−3.669
	SE = 1.025	SE = 1.065
	p = 0.018	p = 0.001
Anti-state attitudes	−0.037	−0.514
	SE = 0.175	SE = 0.182
	p = 0.834	p = 0.005
Contact with LGBT individuals	11.972	10.109
	SE = 0.612	SE = 0.635
	p = 0.000	p = 0.000
Party ID	−1.351	−1.702
	SE = 0.188	SE = 0.195
	p = 0.000	p = 0.000
Ideology	−3.048	−3.655
	SE = 0.286	SE = 0.298
	p = 0.000	p = 0.000
White	2.910	1.520
	SE = 0.688	SE = 0.714
	p = 0.000	p = 0.033
Age	−0.128	−0.106
	SE = 0.017	SE = 0.018
	p = 0.000	p = 0.000
Female	5.766	5.719
	SE = 0.580	SE = 0.602
	p = 0.000	p = 0.000
LGB	7.368	6.851
	SE = 1.172	SE = 1.218
	p = 0.000	p = 0.000
Education	1.025	1.099
	SE = 0.277	SE = 0.287
	p = 0.000	p = 0.000
Family income	0.185	0.125
	SE = 0.050	SE = 0.051
	p = 0.000	p = 0.015
Evangelical Protestant	−7.277	−5.145
	SE = 0.805	SE = 0.832
	p = 0.000	p = 0.000
Church attendance	−2.336	−2.239
	SE = 0.206	SE = 0.214
	p = 0.000	p = 0.000
Midwest	−1.113	0.266
	SE = 0.921	SE = 0.958
	p = 0.227	p = 0.781
South	−1.187	−1.268
	SE = 0.843	SE = 0.876
	p = 0.159	p = 0.148
West	0.419	0.264
	SE = 0.916	SE = 0.951
	p = 0.647	p = 0.781
	p = 0.209	p = 0.005
Num.Obs.	6472	6459
R2	0.296	0.303
R2 Adj.	0.293	0.301
AIC	61 402.8	61 751.6
BIC	61 558.6	61 907.4
Log.Lik.	−30 678.399	−30 852.806
F	128.919	133.510
RMSE	22.87	23.72

OLS Estimates of Relationship Between Place Identity and Support for LGBT Rights (Interactive Effects for Sense of Rural Belonging)

	Model 1
(Intercept)	1.017
	SE = 0.022
	p = 0.000
Place identity	0.000
	SE = 0.006
	p = 0.975
Sense of rural belonging	0.008
	SE = 0.005
	p = 0.129
Place identity × sense of rural belonging	−0.006
	SE = 0.002
	p = 0.005
Suburb	0.016
	SE = 0.008
	p = 0.035
Small town	0.018
	SE = 0.009
	p = 0.042
Rural area	−0.004
	SE = 0.011
	p = 0.722
Anti-state attitudes	−0.014
	SE = 0.002
	p = 0.000
Contact with LGBT individuals	0.083
	SE = 0.006
	p = 0.000
Party ID	−0.023
	SE = 0.002
	p = 0.000
Ideology (Conservative)	−0.053
	SE = 0.003
	p = 0.000
White	0.042
	SE = 0.007
	p = 0.000
Age	−0.001
	SE = 0.000
	p = 0.000
Female	0.033
	SE = 0.006
	p = 0.000
LGB	0.040
	SE = 0.012
	p = 0.001
Education	0.010
	SE = 0.003
	p = 0.000
Family income	0.003
	SE = 0.001
	p = 0.000
Evangelical Protestant	−0.096
	SE = 0.008
	p = 0.000
Church attendance	−0.036
	SE = 0.002
	p = 0.000
Midwest	−0.027
	SE = 0.009
	p = 0.005
South	−0.033
	SE = 0.009
	p = 0.000
West	−0.027
	SE = 0.009
	p = 0.005
Num.Obs.	6311
R2	0.430
R2 Adj.	0.428
AIC	1888.9
BIC	2044.1
Log.Lik.	−921.426
F	225.797
RMSE	0.23

C Collapsing "Rural" and "Small-Town" into a Single Measure of Rural Identity

C.1 Group-Based Affect

OLS Estimates of Relationship Between Rural/Small-Town Identity and Group-Based Affect

	Gays and lesbians	Transgender individuals
(Intercept)	79.337	80.039
	SE = 1.669	SE = 1.733
	p = 0.000	p = 0.000
Rural/small-town identity	−2.601	−3.010
	SE = 0.706	SE = 0.733
	p = 0.000	p = 0.000
Suburb	−0.574	−0.989
	SE = 0.755	SE = 0.784
	p = 0.447	p = 0.207
Small town	0.683	1.597
	SE = 0.875	SE = 0.908
	p = 0.435	p = 0.078
Rural area	−3.375	−4.649
	SE = 0.988	SE = 1.026
	p = 0.001	p = 0.000
Anti-state attitudes	−0.048	−0.516
	SE = 0.175	SE = 0.182
	p = 0.783	p = 0.005
Contact with LGBT individuals	11.979	10.130
	SE = 0.613	SE = 0.636
	p = 0.000	p = 0.000
Party ID	−1.372	−1.726
	SE = 0.188	SE = 0.196
	p = 0.000	p = 0.000
Ideology (Conservative)	−3.086	−3.717
	SE = 0.286	SE = 0.298
	p = 0.000	p = 0.000
White	2.760	1.368
	SE = 0.687	SE = 0.713
	p = 0.000	p = 0.055
Age	−0.128	−0.107
	SE = 0.017	SE = 0.018
	p = 0.000	p = 0.000
Female	5.797	5.781
	SE = 0.580	SE = 0.603
	p = 0.000	p = 0.000
LGB	7.393	6.824
	SE = 1.173	SE = 1.220
	p = 0.000	p = 0.000
Education	1.049	1.141
	SE = 0.277	SE = 0.287
	p = 0.000	p = 0.000
Family income	0.191	0.133
	SE = 0.050	SE = 0.051
	p = 0.000	p = 0.010
Evangelical Protestant	−7.412	−5.343
	SE = 0.804	SE = 0.832
	p = 0.000	p = 0.000
Church attendance	−2.358	−2.272
	SE = 0.206	SE = 0.214
	p = 0.000	p = 0.000
Midwest	−1.215	0.120
	SE = 0.918	SE = 0.956
	p = 0.186	p = 0.900
South	−1.279	−1.365
	SE = 0.843	SE = 0.876
	p = 0.129	p = 0.119
West	0.468	0.321
	SE = 0.912	SE = 0.948
	p = 0.608	p = 0.735
Num.Obs.	6472	6459
R2	0.294	0.301
R2 Adj.	0.292	0.299
AIC	61 412.8	61 770.0
BIC	61 555.1	61 912.2
Log.Lik.	−30 685.396	−30 864.004
F	141.493	145.925
RMSE	22.89	23.76

C.2 Support for LGBT rights

OLS Estimates of Relationship Between Rural/Small-Town Identity and Support for LGBT Rights

	LGBT rights index
(Intercept)	1.022
	SE = 0.017
	p = 0.000
Rural/small town identity	−0.022
	SE = 0.007
	p = 0.002
Suburb	0.013
	SE = 0.008
	p = 0.102
Small town	0.014
	SE = 0.009
	p = 0.107
Rural area	−0.016
	SE = 0.010
	p = 0.115
Anti-state attitudes	−0.014
	SE = 0.002
	p = 0.000
Contact with LGBT individuals	0.084
	SE = 0.006
	p = 0.000
Party ID	−0.024
	SE = 0.002
	p = 0.000
Ideology (Conservative)	−0.054
	SE = 0.003
	p = 0.000
White	0.040
	SE = 0.007
	p = 0.000
Age	−0.001
	SE = 0.000
	p = 0.000
Female	0.034
	SE = 0.006
	p = 0.000
LGB	0.040
	SE = 0.012
	p = 0.001
Education	0.011
	SE = 0.003
	p = 0.000
Family income	0.003
	SE = 0.001
	p = 0.000
Evangelical Protestant	−0.098
	SE = 0.008
	p = 0.000
Church attendance	−0.036
	SE = 0.002
	p = 0.000
Midwest	−0.028
	SE = 0.009
	p = 0.003
South	−0.035
	SE = 0.009
	p = 0.000
West	−0.027
	SE = 0.009
	p = 0.005
Num.Obs.	6311
R2	0.428
R2 Adj.	0.426
AIC	1910.6
BIC	2052.4
Log.Lik.	−934.309
F	247.278
RMSE	0.23

C.3 Rural/Small-Town Identity Moderated by Sense of Rural Belonging

OLS Estimates of Relationship Between Rural/Small-Town Identity and Group-Based Affect (Interactive Effects for Sense of Rural Belonging)

	Gays and lesbians	Transgender individuals
(Intercept)	80.316	81.035
	SE = 1.876	SE = 1.947
	p = 0.000	p = 0.000
Rural/small town identity	−2.108	−1.217
	SE = 1.294	SE = 1.344
	p = 0.103	p = 0.365
Sense of rural belonging	−0.366	−0.372
	SE = 0.321	SE = 0.334
	p = 0.254	p = 0.265
Rural/small-town identity × sense of rural belonging	−0.108	−0.606
	SE = 0.461	SE = 0.479
	p = 0.815	p = 0.206
Suburb	−0.652	−1.073
	SE = 0.757	SE = 0.786
	p = 0.389	p = 0.172
Small town	0.648	1.555
	SE = 0.875	SE = 0.908
	p = 0.459	p = 0.087
Rural area	−3.283	−4.428
	SE = 0.991	SE = 1.030
	p = 0.001	p = 0.000
Anti-state attitudes	−0.059	−0.532
	SE = 0.175	SE = 0.182
	p = 0.736	p = 0.003
Contact with LGBT individuals	11.944	10.084
	SE = 0.613	SE = 0.636
	p = 0.000	p = 0.000
Party ID	−1.375	−1.727
	SE = 0.188	SE = 0.196
	p = 0.000	p = 0.000
Ideology (Conservative)	−3.069	−3.680
	SE = 0.286	SE = 0.298
	p = 0.000	p = 0.000
White	2.746	1.375
	SE = 0.687	SE = 0.713
	p = 0.000	p = 0.054
Age	−0.130	−0.108
	SE = 0.017	SE = 0.018
	p = 0.000	p = 0.000
Female	5.803	5.758
	SE = 0.581	SE = 0.603
	p = 0.000	p = 0.000
LGB	7.440	6.884
	SE = 1.173	SE = 1.220
	p = 0.000	p = 0.000
Education	1.030	1.105
	SE = 0.277	SE = 0.287
	p = 0.000	p = 0.000
Family income	0.188	0.128
	SE = 0.050	SE = 0.051
	p = 0.000	p = 0.012
Evangelical Protestant	−7.389	−5.263
	SE = 0.805	SE = 0.833
	p = 0.000	p = 0.000
Church attendance	−2.342	−2.246
	SE = 0.206	SE = 0.214
	p = 0.000	p = 0.000
Midwest	−1.287	0.065
	SE = 0.921	SE = 0.959
	p = 0.163	p = 0.946
South	−1.321	−1.409
	SE = 0.843	SE = 0.876
	p = 0.117	p = 0.108
West	0.343	0.168
	SE = 0.916	SE = 0.952
	p = 0.708	p = 0.860
Num.Obs.	6472	6459
R2	0.294	0.302
R2 Adj.	0.292	0.300
AIC	61 413.4	61 764.6
BIC	61 569.2	61 920.4
Log.Lik.	−30 683.707	−30 859.306
F	128.205	132.625
RMSE	22.89	23.75

OLS Estimates of Relationship Between Rural/Small-Town Identity and Support for LGBT Rights (Interactive Effects for Sense of Rural Belonging)

	LGBT rights index
(Intercept)	81.035
	SE = 1.947
	p = 0.000
Rural/small-town identity	−1.217
	SE = 1.344
	p = 0.365
Sense of rural belonging	−0.372
	SE = 0.334
	p = 0.265
Rural/small town identity × sense of rural belonging	−0.606
	SE = 0.479
	p = 0.206
Suburb	−1.073
	SE = 0.786
	p = 0.172
Small town	1.555
	SE = 0.908
	p = 0.087
Rural area	−4.428
	SE = 1.030
	p = 0.000
Anti-state attitudes	−0.532
	SE = 0.182
	p = 0.003
Contact with LGBT individuals	10.084
	SE = 0.636
	p = 0.000
Party ID	−1.727
	SE = 0.196
	p = 0.000
Ideology	−3.680
	SE = 0.298
	p = 0.000
White	1.375
	SE = 0.713
	p = 0.054
Age	−0.108
	SE = 0.018
	p = 0.000
Female	5.758
	SE = 0.603
	p = 0.000
LGB	6.884
	SE = 1.220
	p = 0.000
Education	1.105
	SE = 0.287
	p = 0.000
Family income	0.128
	SE = 0.051
	p = 0.012
Evangelical Protestant	−5.263
	SE = 0.833
	p = 0.000
Church attendance	−2.246
	SE = 0.214
	p = 0.000
Midwest	0.065
	SE = 0.959
	p = 0.946
South	−1.409
	SE = 0.876
	p = 0.108
West	0.168
	SE = 0.952
	p = 0.860
Num.Obs.	6459
R2	0.302
R2 Adj.	0.300
AIC	61 764.6
BIC	61 920.4
Log.Lik.	−30 859.306
F	132.625
RMSE	23.75

D Testing the Relationship Between Rural Identity and Group-Based Affect

OLS Estimates of Relationship Between Rural Identity and Group-Based Affect

	Gays and lesbians	Transgender individuals
(Intercept)	79.309	79.935
	SE = 1.666	SE = 1.732
	p = 0.000	p = 0.000
Rural identity	−4.086	−3.767
	SE = 0.837	SE = 0.870
	p = 0.000	p = 0.000
Suburb	−0.625	−1.013
	SE = 0.754	SE = 0.784
	p = 0.407	p = 0.196
Small town	−0.189	0.528
	SE = 0.816	SE = 0.848
	p = 0.816	p = 0.533
Rural area	−2.891	−4.504
	SE = 0.991	SE = 1.032
	p = 0.004	p = 0.000
Anti-state attitudes	−0.025	−0.499
	SE = 0.175	SE = 0.182
	p = 0.888	p = 0.006
Contact with LGBT individuals	12.004	10.165
	SE = 0.612	SE = 0.636
	p = 0.000	p = 0.000
Party ID	−1.372	−1.733
	SE = 0.188	SE = 0.195
	p = 0.000	p = 0.000
Ideology (Conservative)	−3.096	−3.736
	SE = 0.286	SE = 0.298
	p = 0.000	p = 0.000
White	2.653	1.210
	SE = 0.683	SE = 0.710
	p = 0.000	p = 0.088
Age	−0.129	−0.108
	SE = 0.017	SE = 0.018
	p = 0.000	p = 0.000
Female	5.731	5.734
	SE = 0.580	SE = 0.603
	p = 0.000	p = 0.000
LGB	7.441	6.911
	SE = 1.171	SE = 1.219
	p = 0.000	p = 0.000
Education	1.040	1.154
	SE = 0.276	SE = 0.287
	p = 0.000	p = 0.000
Family income	0.187	0.129
	SE = 0.050	SE = 0.051
	p = 0.000	p = 0.012
Evangelical Protestant	−7.280	−5.290
	SE = 0.805	SE = 0.832
	p = 0.000	p = 0.000
Church attendance	−2.369	−2.288
	SE = 0.206	SE = 0.214
	p = 0.000	p = 0.000
Midwest	−1.153	0.153
	SE = 0.918	SE = 0.956
	p = 0.209	p = 0.873
South	−1.263	−1.382
	SE = 0.842	SE = 0.876
	p = 0.134	p = 0.115
West	0.320	0.162
	SE = 0.911	SE = 0.948
	p = 0.726	p = 0.865
Num.Obs.	6472	6459
R2	0.295	0.301
R2 Adj.	0.293	0.299
AIC	61 402.5	61 768.1
BIC	61 544.8	61 910.4
Log.Lik.	−30 680.261	−30 863.072
F	142.257	146.065
RMSE	22.87	23.76

E Testing the Relationship Between Rural Identity and Support for LGBT Rights

OLS Estimates of Relationship Between Rural Identity and Support for LGBT Rights

	Model 1
(Intercept)	1.023<br>SE = 0.017<br>p = 0.000
Rural identity	−0.042<br>SE = 0.009<br>p = 0.000
Suburb	0.012<br>SE = 0.008<br>p = 0.116
Small town	0.008<br>SE = 0.008<br>p = 0.366
Rural area	−0.008<br>SE = 0.010<br>p = 0.415
Anti-state attitudes	−0.014<br>SE = 0.002<br>p = 0.000
Contact with LGBT individuals	0.084<br>SE = 0.006<br>p = 0.000
Party ID	−0.024<br>SE = 0.002<br>p = 0.000
Ideology (Conservative)	−0.054<br>SE = 0.003<br>p = 0.000
White	0.040<br>SE = 0.007<br>p = 0.000
Age	−0.001<br>SE = 0.000<br>p = 0.000
Female	0.033<br>SE = 0.006<br>p = 0.000
LGB	0.041<br>SE = 0.012<br>p = 0.001
Education	0.011<br>SE = 0.003<br>p = 0.000
Family income	0.003<br>SE = 0.001<br>p = 0.000
Evangelical Protestant	−0.097<br>SE = 0.008<br>p = 0.000
Church attendance	−0.036<br>SE = 0.002<br>p = 0.000
Midwest	−0.028<br>SE = 0.009<br>p = 0.004
South	−0.034<br>SE = 0.009<br>p = 0.000
West	−0.028<br>SE = 0.009<br>p = 0.003
Num.Obs.	6311
R2	0.429
R2 Adj.	0.427
AIC	1895.8
BIC	2037.5
Log.Lik.	−926.882
F	248.641
RMSE	0.23

F Testing the Relationship Between Rural Identity and Support for LGBTQ Rights (Individual Items)

OLS Estimates of Relationship Between Rural Identity and Support for LGBT Rights (Individual Items)

	Services	Bathrooms	Discrimination laws	Adoption	Same-sex marriage
(Intercept)	2.276	1.444	1.142	1.854	2.164
	SE = 0.104	SE = 0.104	SE = 0.121	SE = 0.118	SE = 0.111
	p = 0.000	p = 0.000	p = 0.000	p = 0.000	p = 0.000
Rural identity	−0.080	−0.247	−0.122	−0.193	−0.127
	SE = 0.051	SE = 0.052	SE = 0.057	SE = 0.055	SE = 0.052
	p = 0.113	p = 0.000	p = 0.032	p = 0.000	p = 0.015
Suburb	0.033	0.071	0.056	0.062	−0.036
	SE = 0.046	SE = 0.047	SE = 0.061	SE = 0.056	SE = 0.051
	p = 0.468	p = 0.132	p = 0.354	p = 0.267	p = 0.487
Small town	0.039	0.004	−0.099	0.108	−0.044
	SE = 0.049	SE = 0.050	SE = 0.060	SE = 0.058	SE = 0.053
	p = 0.425	p = 0.938	p = 0.101	p = 0.063	p = 0.412
Rural area	0.055	−0.105	−0.163	−0.049	0.031
	SE = 0.060	SE = 0.062	SE = 0.070	SE = 0.067	SE = 0.064
	p = 0.358	p = 0.088	p = 0.020	p = 0.471	p = 0.624
Anti-state attitudes	−0.132	−0.052	0.002	−0.033	−0.022
	SE = 0.010	SE = 0.011	SE = 0.013	SE = 0.012	SE = 0.011
	p = 0.000	p = 0.000	p = 0.899	p = 0.006	p = 0.048
Contact with LGBT individuals	0.125	0.261	0.427	0.357	0.457
	SE = 0.037	SE = 0.037	SE = 0.043	SE = 0.041	SE = 0.038
	p = 0.001	p = 0.000	p = 0.000	p = 0.000	p = 0.000
Party ID	−0.119	−0.105	−0.042	−0.052	−0.074
	SE = 0.011	SE = 0.011	SE = 0.014	SE = 0.013	SE = 0.012
	p = 0.000	p = 0.000	p = 0.002	p = 0.000	p = 0.000
Ideology (Conservative)	−0.146	−0.304	−0.195	−0.224	−0.284
	SE = 0.017	SE = 0.018	SE = 0.022	SE = 0.021	SE = 0.019
	p = 0.000	p = 0.000	p = 0.000	p = 0.000	p = 0.000
White	−0.099	0.195	0.228	0.393	0.400
	SE = 0.041	SE = 0.043	SE = 0.052	SE = 0.050	SE = 0.046
	p = 0.016	p = 0.000	p = 0.000	p = 0.000	p = 0.000
Age	−0.004	0.000	0.004	−0.006	−0.009
	SE = 0.001	SE = 0.001	SE = 0.001	SE = 0.001	SE = 0.001
	p = 0.001	p = 0.795	p = 0.003	p = 0.000	p = 0.000
Female	0.099	0.106	0.098	0.237	0.069
	SE = 0.035	SE = 0.036	SE = 0.043	SE = 0.041	SE = 0.038
	p = 0.005	p = 0.004	p = 0.024	p = 0.000	p = 0.073
LGB	0.247	0.629	−0.051	0.191	0.136
	SE = 0.075	SE = 0.086	SE = 0.101	SE = 0.113	SE = 0.098
	p = 0.001	p = 0.000	p = 0.613	p = 0.090	p = 0.165
Education	−0.019	0.102	0.106	0.082	0.076
	SE = 0.017	SE = 0.017	SE = 0.021	SE = 0.020	SE = 0.018
	p = 0.263	p = 0.000	p = 0.000	p = 0.000	p = 0.000
Family income	−0.007	0.008	0.020	0.027	0.021
	SE = 0.003	SE = 0.003	SE = 0.004	SE = 0.003	SE = 0.003
	p = 0.014	p = 0.006	p = 0.000	p = 0.000	p = 0.000
Evangelical Protestant	−0.410	−0.256	−0.191	−0.389	−0.432
	SE = 0.050	SE = 0.050	SE = 0.053	SE = 0.049	SE = 0.049
	p = 0.000	p = 0.000	p = 0.000	p = 0.000	p = 0.000
Church attendance	−0.130	−0.078	−0.040	−0.161	−0.223
	SE = 0.013	SE = 0.013	SE = 0.014	SE = 0.013	SE = 0.013
	p = 0.000	p = 0.000	p = 0.005	p = 0.000	p = 0.000
Midwest	−0.245	−0.159	0.142	−0.123	−0.138
	SE = 0.056	SE = 0.057	SE = 0.071	SE = 0.068	SE = 0.061
	p = 0.000	p = 0.005	p = 0.044	p = 0.071	p = 0.023
South	−0.289	−0.141	0.032	−0.089	−0.095
	SE = 0.051	SE = 0.052	SE = 0.063	SE = 0.063	SE = 0.056
	p = 0.000	p = 0.007	p = 0.613	p = 0.155	p = 0.091
West	−0.295	−0.106	0.050	−0.104	0.022
	SE = 0.056	SE = 0.058	SE = 0.071	SE = 0.070	SE = 0.063
	p = 0.000	p = 0.067	p = 0.482	p = 0.136	p = 0.723
Num.Obs.	6505	6392	6516	6498	6511
AIC	6914.3	6548.5	4339.5	4972.2	5855.0
BIC	7049.9	6683.8	4475.1	5107.8	5990.6
Log.Lik.	−3437.141	−3254.269	−2149.747	−2466.083	−2907.478
RMSE	1.04	1.02	0.82	0.88	0.96

G Testing for Moderation Between Rural ID and Sense of Rural Belonging (Group-Based Affect)

OLS Estimates of Relationship Between Rural Identity and Group-Based Affect (Interactive Effects for Sense of Rural Belonging)

	Gays and lesbians	Transgender individuals
(Intercept)	79.677<br>SE = 1.818<br>p = 0.000	80.374<br>SE = 1.887<br>p = 0.000
Rural identity	−1.689<br>SE = 1.782<br>p = 0.343	1.918<br>SE = 1.853<br>p = 0.301
Sense of rural belonging	−0.154<br>SE = 0.265<br>p = 0.562	−0.204<br>SE = 0.275<br>p = 0.457
Rural identity × sense of rural belonging	−0.744<br>SE = 0.547<br>p = 0.173	−1.808<br>SE = 0.568<br>p = 0.001
Suburb	−0.667<br>SE = 0.756<br>p = 0.377	−1.082<br>SE = 0.784<br>p = 0.168
Small town	−0.182<br>SE = 0.816<br>p = 0.823	0.528<br>SE = 0.847<br>p = 0.533
Rural area	−2.787<br>SE = 0.993<br>p = 0.005	−4.265<br>SE = 1.032<br>p = 0.000
Anti-state attitudes	−0.036<br>SE = 0.175<br>p = 0.837	−0.523<br>SE = 0.182<br>p = 0.004
Contact with LGBT individuals	11.976<br>SE = 0.612<br>p = 0.000	10.109<br>SE = 0.636<br>p = 0.000
Party ID	−1.371<br>SE = 0.188<br>p = 0.000	−1.731<br>SE = 0.195<br>p = 0.000
Ideology (Conservative)	−3.076<br>SE = 0.286<br>p = 0.000	−3.691<br>SE = 0.297<br>p = 0.000
White	2.681<br>SE = 0.684<br>p = 0.000	1.285<br>SE = 0.710<br>p = 0.070
Age	−0.130<br>SE = 0.017<br>p = 0.000	−0.109<br>SE = 0.018<br>p = 0.000
Female	5.723<br>SE = 0.580<br>p = 0.000	5.702<br>SE = 0.603<br>p = 0.000
LGB	7.500<br>SE = 1.171<br>p = 0.000	7.037<br>SE = 1.218<br>p = 0.000
Education	1.024<br>SE = 0.277<br>p = 0.000	1.122<br>SE = 0.287<br>p = 0.000
Family income	0.187<br>SE = 0.050<br>p = 0.000	0.129<br>SE = 0.051<br>p = 0.012
Evangelical Protestant	−7.229<br>SE = 0.805<br>p = 0.000	−5.168<br>SE = 0.832<br>p = 0.000
Church attendance	−2.355<br>SE = 0.206<br>p = 0.000	−2.263<br>SE = 0.214<br>p = 0.000
Midwest	−1.170<br>SE = 0.920<br>p = 0.203	0.157<br>SE = 0.957<br>p = 0.870
South	−1.272<br>SE = 0.842<br>p = 0.131	−1.384<br>SE = 0.875<br>p = 0.114
West	0.273<br>SE = 0.915<br>p = 0.765	0.100<br>SE = 0.950<br>p = 0.916
Num.Obs.	6472	6459
R2	0.296	0.303
R2 Adj.	0.293	0.301
AIC	61 402.7	61 755.2
BIC	61 558.5	61 911.0
Log.Lik.	−30 678.333	−30 854.619
F	128.928	133.263
RMSE	22.87	23.73

H Testing for Moderation Between Rural ID and Sense of Rural Belonging (Support for LGBTQ Rights)

OLS Estimates of Relationship Between Rural Identity and Support for LGBT Rights (Interactive Effects for Sense of Rural Belonging)

	LGBT rights index
(Intercept)	1.028
	SE = 0.019
	p = 0.000
Rural identity	−0.006
	SE = 0.018
	p = 0.728
Sense of rural belonging	−0.002
	SE = 0.003
	p = 0.416
Rural identity × sense of rural belonging	−0.011
	SE = 0.006
	p = 0.044
Suburb	0.011
	SE = 0.008
	p = 0.137
Small town	0.008
	SE = 0.008
	p = 0.354
Rural area	−0.007
	SE = 0.010
	p = 0.489
Anti-state attitudes	−0.014
	SE = 0.002
	p = 0.000
Contact with LGBT individuals	0.084
	SE = 0.006
	p = 0.000
Party ID	−0.024
	SE = 0.002
	p = 0.000
Ideology	−0.054
	SE = 0.003
	p = 0.000
White	0.040
	SE = 0.007
	p = 0.000
Age	−0.001
	SE = 0.000
	p = 0.000
Female	0.033
	SE = 0.006
	p = 0.000
LGB	0.042
	SE = 0.012
	p = 0.000
Education	0.010
	SE = 0.003
	p = 0.000
Family income	0.003
	SE = 0.001
	p = 0.000
Evangelical Protestant	−0.096
	SE = 0.008
	p = 0.000
Church attendance	−0.036
	SE = 0.002
	p = 0.000
Midwest	−0.028
	SE = 0.009
	p = 0.003
South	−0.034
	SE = 0.009
	p = 0.000
West	−0.029
	SE = 0.009
	p = 0.002
Num.Obs.	6311
R2	0.430
R2 Adj.	0.428
AIC	1891.6
BIC	2046.8
Log.Lik.	−922.778
F	225.572
RMSE	0.23
